# The Impact of Age on Preferences for Colorectal Cancer Surveillance Strategies: Are Fecal Immunochemical Tests FIT for Surveillance?

**DOI:** 10.1002/cam4.70723

**Published:** 2025-03-03

**Authors:** Maddison Dix, Sarah Cohen‐Woods, Molla M. Wassie, Jean M. Winter, Carlene J. Wilson, Graeme P. Young, Charles Cock, Erin L. Symonds

**Affiliations:** ^1^ Flinders Health and Medical Research Institute, College of Medicine and Public Health Flinders University Adelaide South Australia Australia; ^2^ Institute for Mental Health and Wellbeing, College of Education, Psychology and Social Work Flinders University Adelaide South Australia Australia; ^3^ Centre for Epidemiology and Biostatistics, Melbourne School of Population and Global Health The University of Melbourne Melbourne Victoria Australia; ^4^ Department of Gastroenterology Flinders Medical Centre Adelaide South Australia Australia

**Keywords:** colonoscopy, colorectal neoplasms, early detection of cancer, fecal occult blood test, patient preference, population surveillance

## Abstract

**Introduction:**

Individuals with a known risk of colorectal cancer (CRC) are recommended regular surveillance colonoscopies. Alternative surveillance strategies incorporating fecal immunochemical tests (FIT) may improve colonoscopy resource utilization and be more appropriate for those with a lower risk of CRC, particularly younger adults. This study compared younger (< 50 years) and older (≥ 50 years) adults' preferences for different CRC surveillance strategies.

**Methods:**

Eight hundred individuals enrolled in a colonoscopy‐based surveillance program were invited to complete a survey assessing CRC surveillance preferences. Preferences for colonoscopy frequency and the acceptability of two alternative protocols were assessed: (1) providing FIT between colonoscopies, and (2) a FIT‐only strategy where colonoscopy would only be required after a positive FIT result.

**Results:**

A total of 102 younger (median age 41.4 years, 67.6% female) and 187 older (median age 68.5 years, 49.2% female) adults completed the survey. Surveillance preferences did not significantly vary by age group; most respondents preferred colonoscopies more often than their current frequency (< 50 years: 54.1%; ≥ 50 years: 58.1%). Although most participants (< 50 years: 91.2%; ≥ 50 years: 93.0%) agreed that FIT is important to complete between surveillance colonoscopies, only a small proportion were comfortable with FIT‐only surveillance replacing colonoscopies (< 50 years: 27.5%; ≥ 50 years: 37.4%). Fear of CRC was a significant predictor of preferences for more frequent surveillance incorporating FIT in younger, but not older, adults.

**Conclusion:**

Many individuals with an elevated risk of CRC wanted more frequent surveillance, regardless of their age. Extending surveillance colonoscopy intervals using FIT may be a more acceptable method of reducing colonoscopy frequency rather than utilizing a FIT‐only approach.

**Trial Registration:**

This study was prospectively registered with the Australian New Zealand Clinical Trials Registry (ACTRN #12619001743156)

## 1 Introduction

Colorectal cancer (CRC) is on the rise in younger adults. The global incidence of young‐onset CRC (cases diagnosed in adults aged under 50 years) more than doubled between 1990 and 2019 [1], with the factors behind this rising incidence largely unknown [2]. Similarly, the prevalence of young‐onset colorectal adenoma, the precursor lesion of most CRCs, has also increased in younger adults from an estimated 4.2% prior to 1995 to 10.0% after 1995 [3]. Although CRC incidence and mortality have reduced in older adults as a result of participation in screening and surveillance programs, CRC continues to increase in adults younger than the CRC screening age, which typically starts at the age of 50 years [4]. Population engagement in prevention strategies is crucial for improving health outcomes, and given the observed increase of young‐onset CRC and adenoma, younger adults' opinions of CRC prevention methods, and whether these attitudes vary from those of older adults, need to be established.

Certain factors, such as a prior history of colorectal adenoma or a significant family history of CRC, increase the risk of CRC development. Consequently, individuals with these risk factors are typically recommended surveillance colonoscopies at 1–10 yearly intervals, dependent on prior pathology and other risk factors [5, 6]. CRC surveillance guidelines are predominantly based on data obtained from older adult cohorts and often do not include age‐specific recommendations for younger adults with neoplastic findings [7, 8, 9]. Additionally, studies assessing the risk of future neoplasia development within surveillance populations have identified that younger adults do not have an increased risk of advanced neoplasia (advanced adenoma or CRC) compared to older adults [10, 11], indicating that colonoscopies may be overutilized in some younger cohorts. This highlights a need to develop personalized surveillance recommendations for younger adults that consider the opinions of those undergoing surveillance.

Considering the current shift internationally towards lowering the starting age for CRC screening to 45 years old [12, 13], it is expected that there will be an influx of younger people who will require ongoing CRC surveillance [14]. Starting surveillance at a younger age will increase the total number of colonoscopies performed over a person's lifetime and place an increased burden on colonoscopy resources in healthcare systems [15]. There is therefore a need for an alternative surveillance method that can safely reduce colonoscopy frequency without increasing the risk of interval cancer development [16].

One approach could be to use fecal immunochemical tests (FIT), a self‐administered fecal sampling test that is commonly used to determine the need for colonoscopy based on the presence of hemoglobin above a set threshold [17]. Findings from a CRC surveillance program offering FIT between surveillance colonoscopies determined that negative FIT results could identify those with lower risks of future advanced neoplasia development, with these effects accumulating over multiple rounds of negative results [18]. We have previously shown, in an older cohort, that there is low acceptability for the idea of FIT replacing surveillance colonoscopies [19]; however, it is currently unknown whether younger adults would be accepting of surveillance incorporating FIT. Younger adults would have less exposure to fecal testing due to their age, and age itself is an important predictor of CRC screening uptake; participation within current population‐based screening programs increases with age [20]. In addition to age, factors including sex [21], attitudes towards different testing methods [19], prior testing experiences [22, 23], and fears of cancer [24] can influence engagement in CRC screening and surveillance. Therefore, the aim of the current study was to compare younger and older adults' preferences for CRC surveillance strategies.

## 2 Methods

### 2.1 Participants and Study Design

This study employed a cross‐sectional survey design to assess CRC surveillance preferences and attitudes among adults enrolled in the Southern Cooperative Program for the Prevention of Colorectal Cancer (SCOOP). SCOOP is a nurse‐led South Australian CRC surveillance program operating in public and private hospitals throughout the Southern Adelaide region. The program provides colonoscopies for individuals with an elevated risk of CRC due to a family history of CRC or a previous finding of colorectal adenoma at intervals consistent with Australian guidelines [7, 25]. Some individuals enrolled in the program receive FIT at 1–2 yearly intervals between scheduled colonoscopies [26], with most of these individuals aged over 50 years. There are currently around 18,000 individuals undergoing CRC surveillance through this program, including approximately 1500 younger adults aged below 50 years.

Eight hundred individuals who had completed at least one prior colonoscopy were selected using stratified random sampling to ensure equal proportions of individuals by age group (< 50 years vs. ≥ 50 years') and healthcare sector (public vs. private). Individuals were eligible for survey participation if they were: (1) older than 18 years of age, (2) enrolled in the SCOOP program, and (3) awaiting a surveillance procedure at the time of their survey response. Higher risk groups were excluded, including those with a prior diagnosis of inflammatory bowel disease, a genetic syndrome, or CRC. Selected individuals were invited to complete an online survey administered using REDCap [27] between September 2022 and February 2023. Individuals were provided a paper copy of the survey to complete upon request, along with a reply‐paid envelope. Reminder letters were sent with an enclosed paper copy of the survey after 8 weeks of no response.

Ethical approval was granted by the Southern Adelaide Clinical Human Research Ethics Committee (approval #307.18) and this study was prospectively registered with the Australian New Zealand Clinical Trials Registry (registration number 12619001743156).

## 3 Measures

### 3.1 Outcome Variables

The primary outcome was participants' preferences for three CRC surveillance strategies. Respondents' preferences were assessed regarding standard practice (surveillance colonoscopy) and two alternative surveillance protocols: (1) providing FIT between surveillance colonoscopies, and (2) a FIT‐only strategy. The survey indicated that a colonoscopy would be required following a positive FIT result for either of the modified protocols. Preferences for colonoscopy frequency and the timing of FIT provided within the two alternative protocols were assessed using one item per protocol that asked participants to select their preferred timing from the following ordinal options: yearly, 2‐yearly, 3‐yearly, 4‐yearly, 5‐yearly, 10‐yearly (the 10‐yearly option was not included for the FIT‐only surveillance protocol), or never. Colonoscopy preferences were compared to each participant's current surveillance interval to determine whether the individual preferred colonoscopy less often, at the same frequency, or more often. For the two FIT surveillance strategies, individuals who selected a more frequent timing option (e.g., every 1 or 2 years) rather than a less frequent timing option (e.g., every 10 years or never) are referred to as having a “frequent” FIT preference.

Acceptability of the alternative surveillance protocols was assessed as a secondary outcome using two items measuring: (1) a belief in the importance of FIT between colonoscopies, and (2) comfort with the FIT‐only surveillance strategy. Importance was assessed using a yes/no response item, whereas comfort was measured using a 5‐point comfortability scale. To assess whether respondents' perceptions of FIT‐only surveillance were more positive, neutral, or negative, comfort was converted into a 3‐point ordinal outcome by categorizing those who were comfortable, neither comfortable nor uncomfortable, and uncomfortable.

### 3.2 Predictor and Covariate Variables

Age and other sociodemographic, clinical, and psychological characteristics (attitudes to surveillance and fear of CRC) were included as potential predictor variables. Sociodemographic and clinical information was collected from self‐reported items within the survey or from the SCOOP clinical database. Variables included: age, sex, healthcare sector (public vs. private), marital status, employment status, highest level of education completed, country of birth, smoking status, time since most recent FIT completed (for those with prior experience), current surveillance colonoscopy interval length, number of prior colonoscopies, time since most recent colonoscopy, indication (surveillance vs. other) and findings (adenoma vs. no adenoma) at most recent colonoscopy, family history of CRC, personal history of colorectal adenoma, and prior history of non‐CRC cancers.

Attitudes to CRC surveillance testing (colonoscopy and FIT) were assessed by measuring respondents' perceived barriers and ability (confidence) to perform each test. Perceived barriers were measured on a 5‐point Likert scale using 5 barriers per test type. This included 3 test‐specific barriers for FIT and colonoscopy (FIT: messy, unhygienic, distasteful; colonoscopy: nausea, painful, costly) as well as 2 barriers relevant to both test types (unpleasant, embarrassing). Similarly, respondents' perceived ability to perform FIT and colonoscopy was assessed on a 5‐point Likert scale using 2 items per test type that measured ease of test use and ease in finding time for test completion. To assess whether peoples' perceptions were more positive, neutral, or negative in ordinal logistic regression analysis, Likert scale responses were converted into a 3‐point ordinal outcome by categorizing those who agreed, neither agreed nor disagreed, and disagreed with the statements.

Fear of CRC was measured using a modified version of the Fear of Cancer Recurrence Inventory—Short Form (FCRI‐SF), a tool that has displayed good psychometric properties within cancer settings [28]. This tool was adapted to measure a general fear of CRC with permission from the first author [29]. Total summed scores were calculated from 9 items measured on a 5‐point scale ranging from 0 (not at all) to 4 (a great deal).

### 3.3 Statistical Analysis

Analyses were performed using Jamovi version 2.3.21 [30], with statistical significance established at *p* values < 0.05. Statistical assumptions were examined prior to analysis, and non‐parametric tests were used for data with violated assumptions. Age‐related differences were initially explored using chi‐square and Mann–Whitney *U* tests. To determine if there were different predictors of surveillance preferences in younger and older adults, univariate and multivariable analyses were conducted separately for the two age groups (< 50 years and ≥ 50 years). Variables determined to have a statistically significant relationship with the three primary outcomes in univariate analysis (Mann–Whitney *U* tests and Spearman correlations) were further examined within multivariable ordinal logistic regression models to assess the robustness of univariate findings. Participant age and sex were included as baseline characteristics throughout all multivariable models, with age included as a continuous variable for all age‐specific analyses (< 50 years vs. ≥ 50 years). Effect sizes were estimated from odds ratios (OR) with a 95% confidence interval (CI).

## 4 Results

The survey achieved an overall response rate of 36.1% (*N* = 289) of the invited cohort. When stratified by age group, 25.5% (*n* = 102) of younger and 46.8% (*n* = 189) of older adults responded to the survey, where a higher proportion of younger adults chose to complete the survey online (76.5% vs. 46.0%, *p* < 0.001). The median age of the younger and older responding cohort was 41.4 years and 68.5 years, respectively. A large proportion of the cohort (83.0%) were currently recommended colonoscopies at five‐year intervals or longer, with approximately two‐thirds of the cohort (66.2%) having precancerous neoplasia (including adenomas and sessile serrated lesions) removed at their most recent colonoscopy. Most of the cohort (80.0%) also reported prior FIT participation. Among those with previous FIT experience (*n* = 230), 70.4% had completed their most recent test within 2 years prior to the survey (Table 1). Compared to the older cohort, a significantly larger proportion of younger adults were female, currently employed, had completed higher levels of education, were born in Australia, had never completed FIT, and had a self‐reported family history of CRC. In contrast, the older cohort was significantly more likely to have a personal history of colorectal adenoma and a prior diagnosis of non‐CRC cancers (Table 1).

**TABLE 1:** Univariate analysis of survey respondents' sociodemographic and clinical factors by age group.

	Full cohort (*N* = 289)		< 50 year group (*n* = 102)		≥ 50 year group (*n* = 187)		*p*
	*N*	%	*n*	%	*n*	%	
Age (years), M (IQR); range	60.7 (45.9–70.4)	23.1–83.7	41.4 (37.1–46.6)	23.1–50.0	68.5 (61.3–73.5)	51.1–83.7	**< 0.001**
Sex							
Male	128	44.3	33	32.4	95	50.8	**0.003**
Female	161	55.7	69	67.6	92	49.2	
Healthcare sector							
Public	148	51.2	50	49.0	98	52.4	0.582
Private	141	48.8	52	51.0	89	47.6	
Marital status^a^							
Married/de facto	204	71.8	66	68.0	138	73.8	0.307
Not married/de facto	80	28.2	31	32.0	49	26.2	
Employment status^a^							
Currently employed	144	51.1	84	87.5	60	32.3	**< 0.001**
Not currently employed	138	48.9	12	12.5	126	67.7	
Highest level of education completed^a^							
Did not finish secondary education	81	28.5	11	11.3	70	37.4	**< 0.001**
Secondary education	40	14.1	14	14.4	26	13.9	
Tertiary certificate/diploma	83	29.2	31	32.0	52	27.8	
Undergraduate degree	34	12.0	16	16.5	18	9.6	
Postgraduate degree	46	16.2	25	25.8	21	11.2	
Country of birth^a^							
Australia	204	72.3	78	80.4	126	68.1	**0.028**
Not Australia	78	27.7	19	19.6	59	31.9	
Smoking status^a^							
Current/former smokers	149	53.8	44	45.8	105	58.0	0.053
Never smoked	128	46.2	52	54.2	76	42.0	
Most recent FIT completed^a^							
< 1 year ago	68	25.3	14	14.7	54	31.0	**< 0.001**
1–2 years ago	94	34.9	26	27.4	68	39.1	
> 2 years ago	68	25.3	22	23.2	46	26.4	
Never	39	14.5	33	34.7	6	3.4	
Current surveillance colonoscopy interval							
1 year	3	1.0	2	2.0	1	0.5	0.829
3 years	46	15.9	15	14.7	31	16.6	
≥ 5 years	240	83.0	85	83.3	155	82.9	
Number of prior colonoscopies, M (IQR); range	2 (1–3)	1–7	1 (1–2)	1–6	2 (1–4)	1–7	**< 0.001**
Time since most recent colonoscopy (years), M (IQR); range	2.10 (1.39–3.27)	0.21–4.72	2.13 (1.39–3.26)	0.21–4.72	2.06 (1.41–3.22)	0.50–4.64	0.911
Indication for most recent colonoscopy							
Surveillance	158	54.7	50	49.0	108	57.8	0.154
Other^b^	131	45.3	52	51.0	79	42.2	
Findings at most recent colonoscopy							
Colorectal adenoma	190	66.2	63	62.4	127	68.3	0.313
No colorectal adenoma^c^	97	33.8	38	37.6	59	31.7	
Personal history of adenoma							
Yes	259	89.6	86	84.3	173	92.5	**0.029**
No	30	10.4	16	15.7	14	7.5	
Family history of CRC^a^							
Yes	126	45.3	53	55.2	73	40.1	**0.016**
No	152	54.7	43	44.8	109	59.9	
Personal history of other cancers^a^							
Yes	42	18.4	4	5.0	38	25.7	**< 0.001**
No	186	81.6	76	95.0	110	74.3	

*Note:* Bold text signifies a statistically significant difference by age after chi‐square analyses for categorical and Mann–Whitney *U* tests for continuous/ordinal variables (*p* < 0.05).

Abbreviations: CRC = colorectal cancer, FIT = fecal immunochemical test, IQR = interquartile range, M = median.

^a^ Variables contained incomplete observations due to missing survey data.

^b^ Other colonoscopy indications included those with symptoms, positive FIT results, and abnormal scans.

^c^ Normal or benign findings at colonoscopy (including hyperplastic polyps < 10 mm). Findings from 2 participants (1 from each age group) were excluded due to having an incomplete procedure (e.g., due to poor bowel preparation).

**FIGURE 1:**
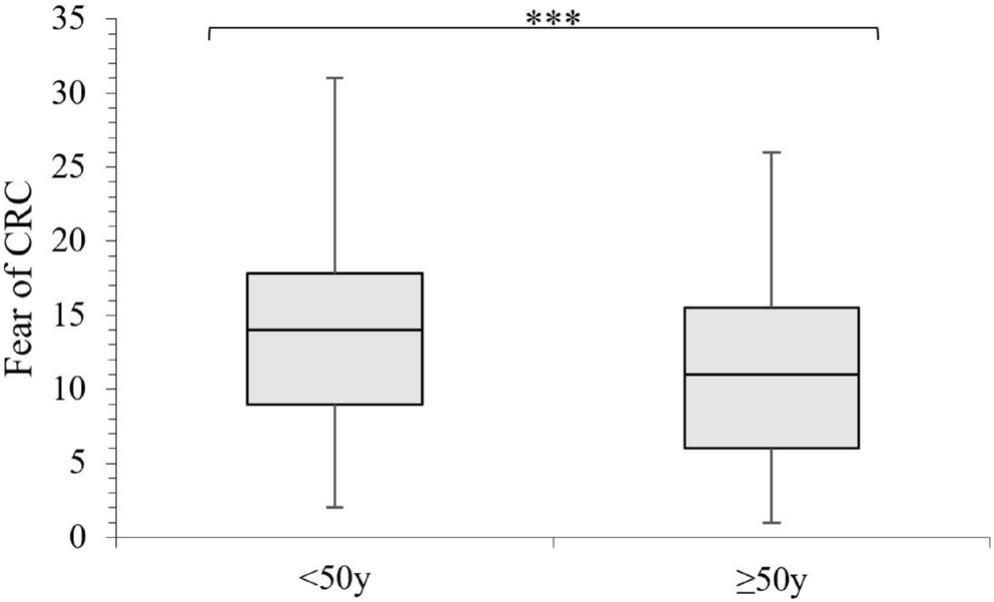
Box and whisker plot of fear of CRC scores by age group. CRC = colorectal cancer, y = years. ***p ≤ 0.001.

Exploration of younger and older adults' psychological characteristics indicated that younger adults had a significantly higher fear of CRC than older adults (Figure 1), and significant age‐related differences were observed in attitudes to FIT and colonoscopy‐related barriers (Table 2). When compared to older adults, the younger cohort was more likely to agree that FIT is unpleasant (28.1% vs. 16.0%) and that colonoscopy caused feelings of nausea (31.6% vs. 14.5%), but they were less likely to disagree that FIT is embarrassing (58.3% vs. 74.9%), messy (28.4% vs. 55.7%), unhygienic (47.9% vs. 73.1%), and distasteful (47.9% vs. 69.7%).

**TABLE 2:** Univariate analysis of attitudes towards colonoscopy and FIT by age group.

Attitudes to colonoscopy					Attitudes to FIT				
	Full cohort *N* (%)	< 50 year group *n* (%)	≥ 50 year group *n* (%)	*p*		Full cohort *N* (%)	< 50 year group *n* (%)	≥ 50 year group *n* (%)	*p*
*Unpleasant*					*Unpleasant*				
Agree	137 (49.1)	51 (51.5)	86 (47.8)	0.302	Agree	55 (20.3)	27 (28.1)	28 (16.0)	**< 0.001**
Neutral	83 (29.7)	32 (32.3)	51 (28.3)		Neutral	102 (37.6)	41 (42.7)	61 (34.9)	
Disagree	59 (21.1)	16 (16.2)	43 (23.9)		Disagree	114 (42.1)	28 (29.2)	86 (49.1)	
*Embarrassing*					*Embarrassing*				
Agree	33 (12.0)	13 (13.4)	20 (11.2)	0.728	Agree	25 (9.2)	10 (10.4)	15 (8.6)	**0.009**
Neutral	81 (29.3)	28 (28.9)	53 (29.6)		Neutral	59 (21.8)	30 (31.3)	29 (16.6)	
Disagree	162 (58.7)	56 (57.7)	106 (59.2)		Disagree	187 (69.0)	56 (58.3)	131 (74.9)	
*Nausea*					*Messy*				
Agree	57 (20.6)	31 (31.6)	26 (14.5)	**0.001**	Agree	61 (22.5)	22 (23.2)	39 (22.2)	**0.002**
Neutral	53 (19.1)	19 (19.4)	34 (19.0)		Neutral	85 (31.4)	46 (48.4)	39 (22.2)	
Disagree	167 (60.3)	48 (49.0)	119 (66.5)		Disagree	125 (46.1)	27 (28.4)	98 (55.7)	
*Painful*					*Unhygienic*				
Agree	13 (4.7)	4 (4.1)	9 (5.1)	0.188	Agree	18 (6.6)	9 (9.4)	9 (5.1)	**< 0.001**
Neutral	37 (13.5)	18 (18.6)	19 (10.7)		Neutral	79 (29.2)	41 (42.7)	38 (21.7)	
Disagree	225 (81.8)	75 (77.3)	150 (84.3)		Disagree	174 (64.2)	46 (47.9)	128 (73.1)	
*Costly*					*Distasteful*				
Agree	44 (16.1)	21 (21.6)	23 (13.0)	0.342	Agree	26 (9.6)	8 (8.3)	18 (10.3)	**0.003**
Neutral	105 (38.3)	33 (34.0)	72 (40.7)		Neutral	77 (28.4)	42 (43.8)	35 (20.0)	
Disagree	125 (45.6)	43 (44.3)	82 (46.3)		Disagree	168 (62.0)	46 (47.9)	122 (69.7)	
*Easy to do*					*Easy to do*				
Agree	221 (77.8)	77 (78.6)	144 (77.4)	0.913	Agree	236 (83.7)	78 (79.6)	158 (85.9)	0.185
Neutral	38 (13.4)	11 (11.2)	27 (14.5)		Neutral	38 (13.5)	17 (17.3)	21 (11.4)	
Disagree	25 (8.8)	10 (10.2)	15 (8.1)			8 (2.8)	3 (3.1)	5 (2.7)	
*Finding time easy*					*Finding time easy*				
Agree	223 (79.1)	71 (73.2)	152 (82.2)	0.058	Agree	238 (84.4)	80 (81.6)	158 (85.9)	0.356
Neutral	44 (15.6)	17 (17.5)	27 (14.6)		Neutral	34 (12.1)	14 (14.3)	20 (10.9)	
Disagree	15 (5.3)	9 (9.3)	6 (3.2)		Disagree	10 (3.5)	4 (4.1)	6 (3.3)	

*Note:* Bold text signifies a statistically significant difference after Mann–Whitney *U* test analyses (*p* < 0.05). All variables contained incomplete observations due to missing survey data.

Abbreviation: FIT = fecal immunochemical test.

### 4.1 Preference for Surveillance Colonoscopy Frequency

**FIGURE 2:**
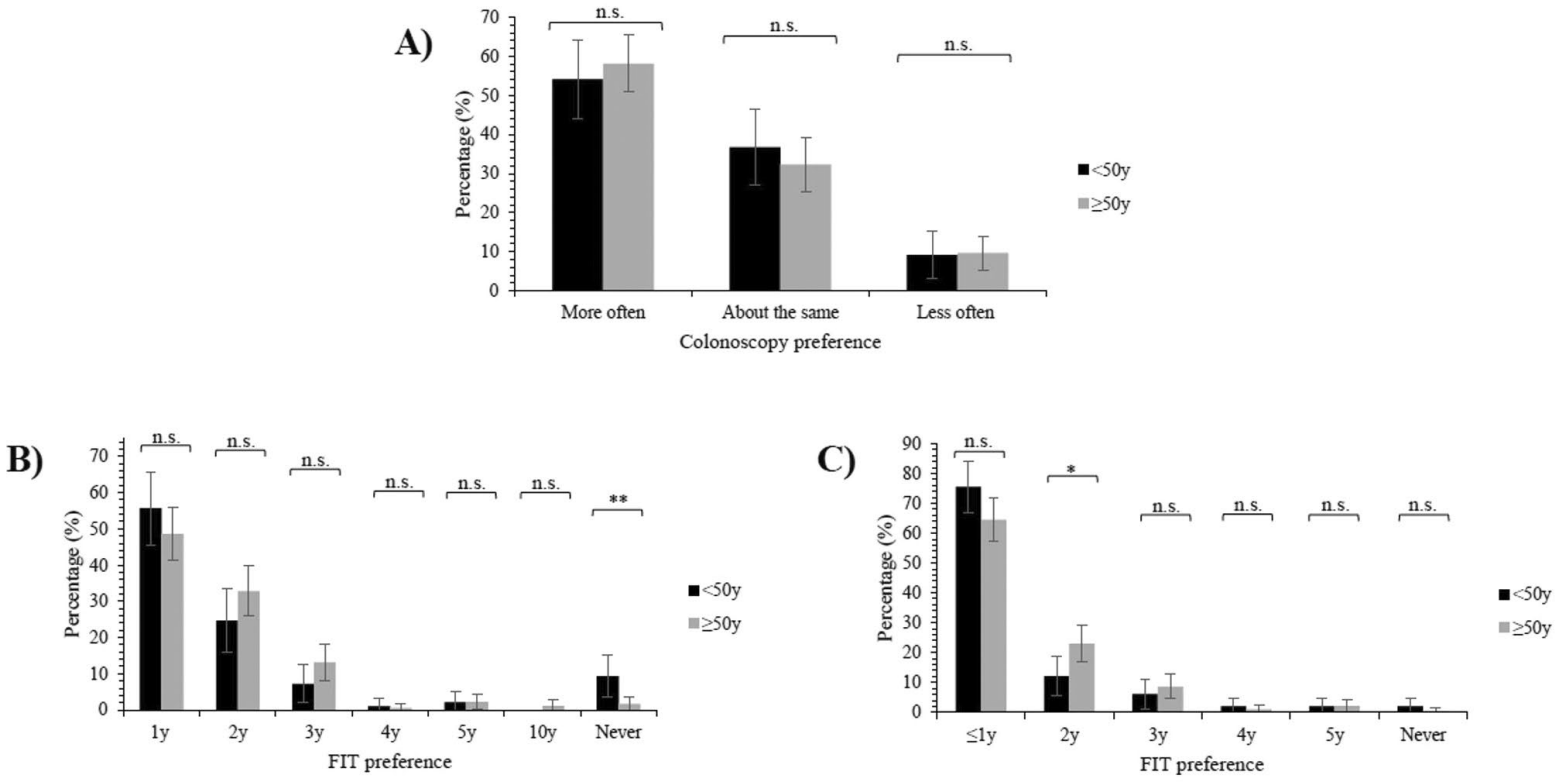
Preferences for CRC surveillance strategies by age group. (A) Surveillance colonoscopy frequency compared to current recommended guidelines, (B) FIT provided between surveillance colonoscopies, and (C) FIT provided without surveillance colonoscopies. FIT = fecal immunochemical test, n.s. = not significant (*p* ≥ 0.05), y = years. *p < 0.05, **p ≤ 0.01.

Respondents' current surveillance colonoscopy interval and preferences for surveillance colonoscopy frequency did not significantly differ by age group. The majority of both younger (54.1%) and older (58.1%) adults (Figure 2A) preferred to have colonoscopies more often than their currently recommended surveillance interval (Table 1).

Throughout both age groups, individuals who had longer surveillance colonoscopy intervals and those with a higher fear of CRC had significantly greater odds of preferring surveillance colonoscopies more often than current recommendations (Table 3). In older adults, those who agreed that colonoscopies are easy to do (OR = 2.37, *p* = 0.025) and those who had completed more prior colonoscopies (OR = 1.30, *p* = 0.043) were also more likely to prefer having colonoscopies more often than their current surveillance interval. Conversely, younger adults with the attitude that colonoscopies are unpleasant (OR = 0.50, *p* = 0.037) and older adults who agreed that colonoscopies are painful (OR = 0.45, *p* = 0.024) were less likely to prefer having colonoscopies more often than their current interval compared to those who did not agree with these attitudes (Table 3).

**TABLE 3:** Multivariable analysis of associations between different factors and a preference for surveillance colonoscopies provided more often than current recommended guidelines.

< 50 year model				≥ 50 year model			
Variable	OR	95% CI	*p*	Variable	OR	95% CI	*p*
Age	1.01	0.94–1.09	0.741	Age	0.96	0.91–1.01	0.093
*Sex*				*Sex*			
Female (male)^a^	1.37	0.54–3.49	0.503	Female (male)^a^	1.03	0.50–2.14	0.935
Current SI^b^	2.07	1.30–3.40	**0.003**	Current SI^b^	1.92	1.20–3.09	**0.007**
Fear of CRC	1.09	1.02–1.18	**0.019**	Fear of CRC	1.15	1.07–1.23	**< 0.001**
Colonoscopy unpleasant^c^	0.50	0.26–0.94	**0.037**	Colonoscopy unpleasant^c^	0.74	0.46–1.19	0.217
Colonoscopy easy to do^c^	1.48	0.67–3.31	0.327	Colonoscopy easy to do^c^	2.37	1.13–5.12	**0.025**
Colonoscopy finding time easy^c^	1.39	0.64–3.03	0.400	Colonoscopy finding time easy^c^	1.07	0.41–2.74	0.894
				*Findings at most recent colonoscopy* ^a^			
				Adenoma (no adenoma)	0.58	0.24–1.33	0.206
				Time since most recent colonoscopy	1.32	0.95–1.85	0.101
				Number of prior colonoscopies	1.30	1.01–1.68	**0.043**
				Education^b^	0.78	0.60–1.00	0.051
				Colonoscopy nausea^c^	0.98	0.57–1.73	0.950
				Colonoscopy painful^c^	0.45	0.22–0.90	**0.024**

*Note:* Bold text signifies a statistically significant difference after multivariable ordinal logistic regression analysis (*p* < 0.05). Colonoscopy preferences were coded in the following order: less often, about the same, more often.

Abbreviations: CI = confidence interval, CRC = colorectal cancer, FIT = fecal immunochemical test, OR = odds ratio, SI = surveillance interval.

^a^ Reference categories are stated in parentheses.

^b^ Ordinal variables were coded in the lowest to highest order (e.g., increasing interval lengths, higher levels of education completed).

^c^ Ordinal variables were coded in the following order: disagree, neither agree nor disagree, agree.

### 4.2 Preference for Including FIT Between Surveillance Colonoscopies

**FIGURE 3:**
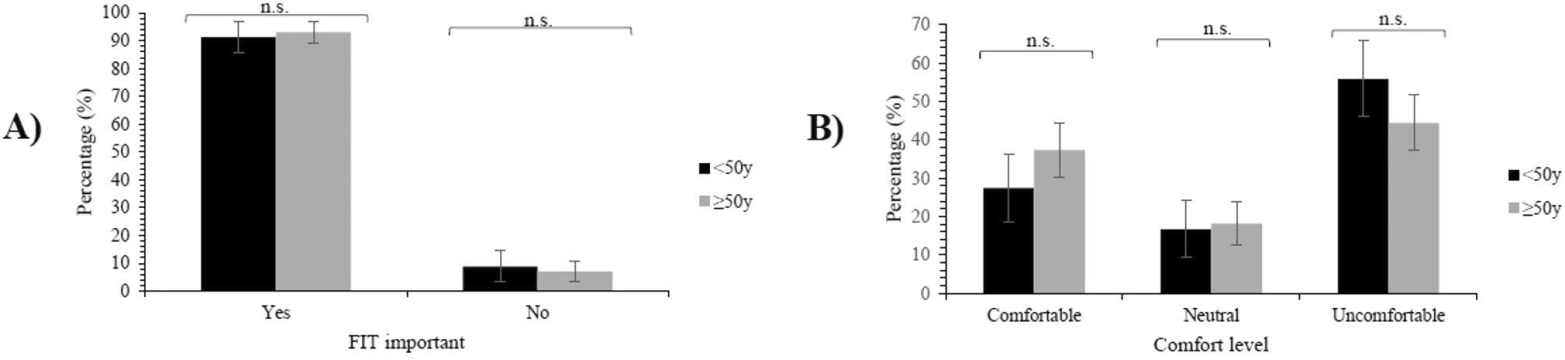
Acceptability of the FIT modified surveillance strategies by age group. (A) belief in the importance of FIT between surveillance colonoscopies, and (B) comfort with FIT‐only surveillance. FIT = fecal immunochemical test, n.s. = not significant (*p* ≥ 0.05), y = years.

Overall, most respondents within both age groups considered it important to complete FIT between surveillance colonoscopies (Figure 3A) and preferred to complete FIT at 1 or 2‐yearly intervals (Figure 2B). Younger adults were more likely to prefer to never complete FIT between surveillance colonoscopies than older adults (9.3% vs. 1.6%; Figure 2B). Preferences did not significantly differ between age groups at any other timing (Figure 2B).

Although no variables were determined to significantly predict older adults' preferences for including FIT between colonoscopies in multivariable analysis (Table 4), four variables significantly predicted younger adults' preferences: age, fear of CRC, level of education, and the perception that FIT is unhygienic. Increasing age among the younger cohort (OR = 1.19, *p* < 0.001), higher fear of CRC scores (OR = 1.13, *p* = 0.005), and having completed higher levels of education (OR = 1.99, *p* = 0.002) were associated with greater odds of preferring frequent (e.g., annual) FIT between surveillance colonoscopies. Conversely, those who considered FIT unhygienic were less likely to prefer having frequent FIT than those with less concern about hygiene (OR = 0.26, p = 0.002; Table 4).

**TABLE 4:** Multivariable analysis of associations between different factors and preferences for frequent FIT when provided between surveillance colonoscopies.

< 50 year model				≥ 50 year model			
Variable	OR	95% CI	*p*	Variable	OR	95% CI	*p*
Age	1.19	1.08–1.32	**< 0.001**	Age	1.00	0.96–1.05	0.895
*Sex*				*Sex*			
Female (male)^a^	0.51	0.15–1.61	0.261	Female (male)^a^	0.81	0.42–1.56	0.533
Most recent FIT completed^b^	0.77	0.45–1.30	0.333	Most recent FIT completed^b^	0.73	0.48–1.09	0.123
Education^b^	1.99	1.31–3.13	**0.002**	Education^b^	1.06	0.83–1.34	0.659
FIT unhygienic^c^	0.26	0.11–0.61	**0.002**	FIT unhygienic^c^	1.20	0.55–2.75	0.661
FIT easy to do^c^	3.70	1.03–15.9	0.057	FIT easy to do^c^	1.51	0.66–3.53	0.328
*Country of birth*				*Smoking status*			
Australia (not Australia)^a^	3.16	0.88–11.8	0.080	Current/former (never)^a^	0.57	0.29–1.10	0.098
Fear of CRC	1.13	1.04–1.24	**0.005**	Colonoscopy painful^c^	0.86	0.42–1.79	0.685
FIT finding time easy^c^	1.86	0.47–6.57	0.344	FIT unpleasant^c^	0.81	0.45–1.48	0.485
				FIT distasteful^c^	1.15	0.58–2.28	0.694
				FIT embarrassing^c^	0.73	0.34–1.53	0.411

*Note:* Bold text signifies a statistically significant difference after multivariable ordinal logistic regression analysis (*p* < 0.05). FIT preferences were coded in the following order: never, 10, 5, 4, 3, 2, 1 years.

Abbreviations: CI = confidence interval, CRC = colorectal cancer, FIT = fecal immunochemical test, OR = odds ratio, y = year.

^a^ Reference categories are stated in parentheses.

^b^ Ordinal variables were coded in the lowest to highest order (e.g., increasing time since/never completing FIT, higher levels of education completed).

^c^ Ordinal variables were coded in the following order: disagree, neither agree nor disagree, agree.

### 4.3 Preference for FIT‐Only Surveillance

Over two‐thirds of the cohort preferred to have annual FIT if their surveillance protocol did not include surveillance colonoscopies (Figure 2C). Although overall preferences for FIT‐only surveillance did not significantly differ by age group, a significantly higher proportion of older adults preferred 2‐yearly FIT‐only surveillance than younger adults (23.0% vs. 12.2%; Figure 2C). Additionally, while the differences observed in comfort levels for FIT‐only surveillance did not reach statistical significance (*p* = 0.051), a larger proportion of younger adults reported being uncomfortable with this type of surveillance than older adults (55.9% vs. 44.4%; Figure 3B).

Among younger adults, preferences for FIT‐only surveillance were significantly predicted by four variables: age, fear of CRC, smoking status, and time since the most recent FIT was completed (Table 5). Although increasing age among the younger cohort (OR = 1.27, *p* < 0.001) and higher fear of CRC scores (OR = 1.20, *p* < 0.001) were associated with greater odds of preferring frequent FIT, being a current/former smoker (OR = 0.07, *p* = 0.001) and having a longer length of time since completing FIT or never completing FIT (OR = 0.44, *p* = 0.026) reduced these odds. In older adults, those who reported a family history of CRC were more likely to choose a frequent FIT timing option when offered without regular surveillance colonoscopies than those who did not report a family history of CRC (OR = 2.42, *p* = 0.043). The effect of family history on surveillance preferences did not significantly differ between individuals who had also experienced a previous adenoma and those with only a family history of CRC (Table S1). Similarly, older adults with a longer length of time since their most recent colonoscopy were more likely to prefer having frequent FIT surveillance (OR = 1.60, *p* = 0.013; Table 5).

**TABLE 5:** Multivariable analysis of associations between different factors and preferences for frequent FIT when provided without surveillance colonoscopies.

< 50 year model				≥ 50 year model			
Variable	OR	95% CI	*p*	Variable	OR	95% CI	*p*
Age	1.27	1.11–1.47	**< 0.001**	Age	0.98	0.92–1.05	0.576
*Sex*				*Sex*			
Female (male)^a^	1.26	0.29–5.06	0.748	Female (male)^a^	0.85	0.39–1.84	0.679
*Family history of CRC*				*Family history of CRC*			
Yes (no)^a^	2.88	0.82–10.9	0.105	Yes (no)^a^	2.42	1.05–5.85	**0.043**
Most recent FIT completed^b^	0.44	0.20–0.87	**0.026**	Most recent FIT completed^b^	0.64	0.40–1.02	0.062
FIT distasteful^c^	2.83	0.76–11.9	0.136	FIT distasteful^c^	0.80	0.38–1.71	0.564
*Smoking status*				*Current employment status*			
Current/former (never)^a^	0.07	0.01–0.31	**0.001**	Employed (not employed)^a^	2.54	0.93–7.31	0.075
Fear of CRC	1.20	1.09–1.35	**< 0.001**	Time since most recent colonoscopy	1.60	1.12–2.36	**0.013**
FIT unhygienic^c^	0.33	0.09–1.08	0.072	Education^b^	1.17	0.89–1.56	0.274
				Colonoscopy painful^c^	0.50	0.23–1.12	0.090
				FIT messy^c^	0.97	0.54–1.76	0.913
				FIT unpleasant^c^	1.28	0.61–2.80	0.532
				FIT embarrassing^c^	1.10	0.57–2.21	0.787
				FIT easy to do^c^	2.06	0.58–7.60	0.265
				FIT finding time easy^c^	1.57	0.50–4.74	0.429

*Note:* Bold text signifies a statistically significant difference after multivariable ordinal logistic regression analysis (*p* < 0.05). FIT preferences were coded in the following order: never, 5, 4, 3, 2, 1 years.

Abbreviations: CI = confidence interval, CRC = colorectal cancer, FIT = fecal immunochemical test, OR = odds ratio.

^a^ Reference categories are stated in parentheses.

^b^ Ordinal variables were coded in the lowest to highest order (e.g., increasing time since/never completing FIT, higher levels of education completed).

^c^ Ordinal variables were coded in the following order: disagree, neither agree nor disagree, agree.

## 5 Discussion

In this study, younger adults' (< 50 years) preferences for surveillance colonoscopy frequency and two alternative protocols incorporating FIT were investigated and compared to the preferences of those aged ≥ 50 years. Overall, surveillance preferences did not significantly vary between younger and older adults; most respondents, regardless of age, wanted more frequent CRC surveillance with both surveillance colonoscopies and FIT within the two alternative protocols. Although almost all respondents (92.4%) believed that FIT is important to complete between surveillance colonoscopies, approximately half of the entire cohort was uncomfortable with a FIT‐only surveillance strategy. Together, these findings demonstrate that (1) a large proportion of individuals undergoing CRC surveillance would prefer to have testing done more frequently than current guideline recommendations, and (2) FIT could be utilized to offer those wanting more frequent surveillance without increasing risks associated with increasing surveillance colonoscopy frequency beyond that of current guideline recommendations [15].

Utilizing FIT throughout CRC surveillance could improve the risk management of individuals undergoing regular colonoscopies by creating personalized surveillance colonoscopy intervals: scheduling colonoscopies earlier for those with positive FIT results and enabling extensions to surveillance colonoscopy intervals following negative results. Findings from the present study indicate that extensions to surveillance colonoscopy intervals using FIT may be a more acceptable method of personalizing CRC surveillance for younger adults who have a lower risk of advanced neoplasia [10, 11], rather than a FIT‐only approach. However, while these results have demonstrated that FIT provided between surveillance colonoscopies is generally accepted by those undergoing CRC surveillance, the acceptability of a protocol that extends surveillance colonoscopy intervals for those with negative FIT results requires further exploration. In addition to FIT, there are a range of alternate CRC screening modalities that could also be used to form personalized recommendations for those undergoing surveillance colonoscopies. This includes a number of non‐invasive testing options that have been approved for clinical use, such as other types of fecal (e.g., multi‐target stool DNA tests) and blood‐based testing options [31]. As test acceptability is likely to vary between these different modalities, with blood‐based screening having equal or higher participation rates in CRC screening cohorts than fecal‐based options [32], future research should determine patients' acceptance of different test types for use in addition to or instead of surveillance colonoscopy. However, as these tools are not currently recommended for use in addition to surveillance colonoscopies, the use of non‐invasive biomarker tests to personalize colonoscopy intervals will first need to be endorsed by clinical guidelines or introduced into policy.

Throughout the present study, fear of CRC was commonly associated with a desire for frequent surveillance using FIT and/or colonoscopy; it was the only variable that was predictive of preferences for all three surveillance strategies in younger adults. Individuals with a higher fear of CRC had greater odds of preferring more frequent CRC surveillance. These findings are consistent with previous research investigating older adults' surveillance preferences after adenoma removal within the English Bowel Cancer Screening Program [22, 23]. These studies reported that individuals undergoing post‐polypectomy surveillance preferred 3‐yearly surveillance colonoscopies over annual FIT due to fears of CRC development. This preference stemmed from beliefs that colonoscopies are more accurate than FIT because they involve visual inspections of the bowel and fears that FIT could miss colorectal lesions that do not bleed or that bleed intermittently [22]. Similar to the present study, a surveillance strategy providing FIT regularly between colonoscopies was more accepted by those receiving colonoscopic surveillance than a FIT‐only protocol that determines the need for follow‐up colonoscopy [23]. Those with a higher fear of CRC within the present study were more likely to prefer having frequent CRC surveillance, suggesting that further research should examine whether fears of cancer influence participation rates in CRC surveillance programs and why certain individuals undergoing surveillance experience higher levels of fear.

In addition to fear of CRC, several of the other sociodemographic, clinical, and psychological factors were predictive of CRC surveillance preferences. Generally, individuals who reported perceived barriers to surveillance were more likely to prefer less frequent surveillance, whereas individuals who were confident in their ability to undergo testing were more likely to prefer more frequent surveillance. These findings are consistent with previous research conducted in CRC screening populations, whereby test participation was associated with lower perceived barriers and higher confidence in ability to undergo testing [33]. Our previous research on attitudes towards FIT‐only surveillance in a separate, older cohort further supported these findings; individuals with higher barriers to colonoscopy had lower confidence in their ability to undergo colonoscopy, which resulted in more comfort with a FIT‐only surveillance strategy that reduces their colonoscopy frequency [19]. Furthermore, the sociodemographic factors identified in this study as being predictive of frequent FIT preferences in the younger cohort (older age, higher levels of education, and never smokers) are consistent with prior research on predictors of FIT uptake within CRC screening programs. Specifically, FIT uptake has been positively associated with older age and higher levels of education, while smoking habits have been linked to poorer participation [34, 35]. These factors highlight key areas for future implementations of surveillance changes to address to ensure optimal uptake of FIT within CRC surveillance populations.

A key strength of the current study was its focus on individuals enrolled within a well‐established CRC surveillance program. Survey responses were collected from ~7% of the younger adult SCOOP population (~1500 individuals), with this sample size being sufficient to estimate the prevalence of surveillance preferences with a ± 10% margin of error [36]. There are, however, certain limitations that should be acknowledged. First, because some participants within the SCOOP program had been offered FIT between surveillance colonoscopies for several years, these individuals may be more accepting of surveillance protocols incorporating FIT than other surveillance populations that only receive colonoscopic surveillance. Second, despite efforts to maximize survey responses by offering online and paper formats, response rates were almost 50% lower in the younger cohort than in older adults. Lastly, fear of CRC was assessed using a modified version of a validated scale, the FCRI‐SF. Although the FCRI‐SF was developed and validated within cancer populations [28, 29], the version used in this study was adapted to measure a general fear of CRC, rather than fears of cancer recurrence. Consequently, the application of this version of the FCRI‐SF to measure fear of CRC should be viewed cautiously as it has not been psychometrically validated for this purpose.

## 6 Conclusion

Findings from the current study indicate that preferences for CRC surveillance strategies do not significantly vary between younger and older adults. Most respondents, regardless of age, wanted more rather than less frequent CRC surveillance. Surveillance incorporating FIT at 1 or 2‐yearly intervals between surveillance colonoscopies was more accepted than a FIT‐only approach that replaces routine colonoscopic surveillance. Therefore, FIT could be provided to individuals wanting more frequent CRC surveillance than current guideline recommendations without increasing colonoscopy demand.

## Author Contributions

**Maddison Dix:** conceptualization (lead), data curation (lead), formal analysis (lead), methodology (equal), project administration (lead), resources (supporting), writing – original draft (lead), writing – review and editing (equal). **Sarah Cohen‐Woods:** conceptualization (supporting), formal analysis (supporting), methodology (equal), supervision (supporting), writing – original draft (supporting), writing – review and editing (equal). **Molla M. Wassie:** conceptualization (supporting), formal analysis (supporting), methodology (supporting), supervision (supporting), writing – original draft (supporting), writing – review and editing (equal). **Jean M. Winter:** project administration (supporting), writing – review and editing (equal). **Carlene J. Wilson:** funding acquisition (equal), methodology (equal), writing – review and editing (equal). **Graeme P. Young:** funding acquisition (equal), writing – review and editing (equal). **Charles Cock:** funding acquisition (supporting), resources (supporting), writing – review and editing (equal). **Erin L. Symonds:** conceptualization (supporting), data curation (supporting), formal analysis (supporting), funding acquisition (equal), methodology (equal), project administration (supporting), resources (lead), supervision (lead), writing – original draft (supporting), writing – review and editing (equal).

## Ethics Statement

Ethics approval was granted by the Southern Adelaide Clinical Human Research Ethics Committee (#307.18).

## Consent

Written informed consent was obtained from all study participants.

## Conflicts of Interest

The authors declare no conflicts of interest.

## Supporting information


**Table S1.** Exploratory analysis of whether having a personal history of adenoma and a family history of CRC affects preferences for frequent FIT when provided without surveillance colonoscopies.

## Data Availability

Deidentified study data are available upon reasonable request from the corresponding author.
