# Supplementary material for: The Impact of Age on Preferences for Colorectal Cancer Surveillance Strategies: Are Fecal Immunochemical Tests FIT for Surveillance?
Source: Cancer Med. 2025 Mar 3;14(5):e70723. doi: 10.1002/cam4.70723 (PMC11873988; doi:10.1002/cam4.70723)
Supplement: Supplementary file 1 — Table S1. Exploratory analysis of whether having a personal history of adenoma and a family history of CRC affects preferences for frequent FIT when provided without surveillance colonoscopies. [file CAM4-14-e70723-s001.docx]

**Supplemental Table 1:** Exploratory analysis of whether having a personal history of adenoma and a family history of CRC affects preferences for frequent FIT when provided without surveillance colonoscopies

| **<50y model** | | | | **≥50y model** | | | |
| --- | --- | --- | --- | --- | --- | --- | --- |
| **Variable** | **OR** | **95% CI** | **p value** | **Variable** | **OR** | **95% CI** | **p value** |
| Age | 1.28 | 1.12-1.49 | **<0.001** | Age | 0.98 | 0.92-1.05 | 0.571 |
| Sex  Female (male)^†^ | 0.96 | 0.23-3.64 | 0.954 | Sex  Female (male)^†^ | 0.82 | 0.38-1.74 | 0.596 |
| Risk status^‡^ | 1.51 | 0.58-3.97 | 0.394 | Risk status^‡^ | 1.95 | 0.99-3.92 | 0.054 |
| Most recent FIT completed^§^ | 0.47 | 0.21-0.93 | **0.041** | Most recent FIT completed^§^ | 0.68 | 0.42-1.08 | 0.104 |
| FIT distasteful^¶^ | 2.57 | 0.67-11.1 | 0.182 | FIT distasteful^¶^ | 0.77 | 0.36-1.65 | 0.500 |
| Smoking status  Current/former (never)^†^ | 0.07 | 0.01-0.29 | **<0.001** | Current employment status  Employed (not employed)^†^ | 2.41 | 0.89-6.81 | 0.088 |
| Fear of CRC | 1.20 | 1.09-1.34 | **<0.001** | Time since most recent colonoscopy | 1.65 | 1.16-2.43 | **0.008** |
| FIT unhygienic^¶^ | 0.34 | 0.09-1.10 | 0.078 | Education^§^ | 1.21 | 0.91-1.61 | 0.193 |
|  | | | | Colonoscopy painful^¶^ | 0.56 | 0.25-1.21 | 0.136 |
|  |  |  |  | FIT messy^¶^ | 0.91 | 0.51-1.64 | 0.748 |
|  |  |  |  | FIT unpleasant^¶^ | 1.39 | 0.66-3.13 | 0.400 |
|  |  |  |  | FIT embarrassing^¶^ | 1.10 | 0.56-2.22 | 0.794 |
|  |  |  |  | FIT easy to do^¶^ | 2.53 | 0.68-9.96 | 0.170 |
|  |  |  |  | FIT finding time easy^¶^ | 1.39 | 0.41-4.43 | 0.587 |

*Note*: Bold text signifies a statistically significant difference after multivariable ordinal logistic regression analysis (p<0.05). FIT preferences were coded in the following order: never, 5y, 4y, 3y, 2y, 1y. OR=odds ratio, CI=confidence interval, y=years, CRC=colorectal cancer, FIT=fecal immunochemical test.

^†^Reference categories are stated in parentheses.

^‡^Risk status was coded as an ordinal variable in the order of increasing risk: family history of CRC only (9.1%, n=26), previous adenoma only (54.0%, n=155), family history of CRC and previous adenoma (36.9%, n=106). Two participants (both from the ≥50y age group) were excluded as their risk status did not match one of the specified categories (one was recommended surveillance after a renal transplant, while the other had only had a prior history of hyperplastic polyps).

^§^Ordinal variables coded in the lowest to highest order (e.g., increasing time since/never completing FIT, higher levels of education completed).

^¶^Ordinal variables coded in the following order: disagree, neither agree nor disagree, agree.
